# Efficacy of a recombinant turkey herpesvirus (H9) vaccine against H9N2 avian influenza virus in chickens with maternal-derived antibodies

**DOI:** 10.3389/fmicb.2022.1107975

**Published:** 2023-01-26

**Authors:** Xue Pan, Qinfang Liu, Shiqi Niu, Dongming Huang, Dawei Yan, Qiaoyang Teng, Xuesong Li, Nancy Beerens, Maria Forlenza, Mart C. M. de Jong, Zejun Li

**Affiliations:** ^1^Shanghai Veterinary Research Institute, Chinese Academy of Agricultural Sciences, Shanghai, China; ^2^Quantitative Veterinary Epidemiology, Animal Sciences Group, Wageningen University and Research, Wageningen, Netherlands; ^3^Wageningen Bioveterinary Research, Wageningen University and Research, Lelystad, Netherlands; ^4^Host-Microbe Interactomics Group, Animal Sciences Group, Wageningen University and Research, Wageningen, Netherlands

**Keywords:** maternal-derived antibodies, passively transferred antibodies, recombinant vaccine, turkey herpesvirus (HVT), immune responses, transmission

## Abstract

Although vaccines have been widely used for many years, they have failed to control H9N2 avian influenza virus (AIV) in the field in China. The high level of maternal-derived antibodies (MDAs) against H9N2 virus contributes to the H9N2 influenza vaccine failure in poultry. The study aimed to generate a new vaccine to overcome MDAs interference in H9N2 vaccination in chickens. We used turkey herpesvirus (HVT) as a vaccine vector to express H9 hemagglutinin (HA) proteins. The recombinant HVT expressing H9 HA proteins (rHVT-H9) was successfully generated and characterized in primary chicken embryonic fibroblasts (CEFs). Western blot and indirect immunofluorescence assay (IFA) showed that the rHVT-H9 consistently expressed HA proteins. In addition, the rHVT-H9 had similar growth kinetics to the parent HVT. Preliminary animal experiments showed that compared to the conventional inactivated whole virus (IWV) vaccine, the rHVT-H9 stimulated robust humoral immunity in chickens with passively transferred antibodies (PTAs) that were used to mimic MDAs. Transmission experiments showed that the rHVT-H9 induced both humoral and cellular immunity in chickens with PTAs. Furthermore, we used mathematical models to quantify the vaccine’s efficacy in preventing the transmission of H9N2 AIV. The results showed that the rHVT-H9 reduced the virus shedding period and decreased the reproduction ratio (R) value in chickens with PTAs after homologous challenge. However, the vaccination in this trial did not yet bring *R* < 1. In summary, we generated a new rHVT-H9 vaccine, which stimulated strong humoral and cellular immunity, reducing virus shedding and transmission of H9N2 AIV even in the presence of PTAs in chickens.

## 1. Introduction

H9N2 avian influenza virus (AIV) is the most widespread and prevalent low pathogenicity avian influenza virus (LPAIV) subtype of influenza virus in the world, posing a serious threat to both the global poultry industry and human health. H9N2 AIV regularly spills over from birds to humans (Burke and Trock, 2018; Pusch and Suarez, 2018). Moreover, it donates partial or even whole sets of internal genes to other influenza viruses such as H5N1 (Guan et al., 2000), H5N6 (Shen et al., 2016), H7N9 (Liu et al., 2013; Bi et al., 2015), H10N8 (Chen et al., 2014; Zhang et al., 2014) and H10N3 (Qi et al., 2022), that can also infect humans, posing a substantial threat to public health.

Vaccination is the main strategy for managing H9N2 AIV in poultry, however maternal-derived antibodies (MDAs) interfere with immune responses contributing to H9N2 vaccination failure. Currently, the H9N2 inactivated whole virus (IWV) vaccine is predominantly used in poultry in many countries, including China (Zhang et al., 2008), Israel (Banet-Noach et al., 2007), South Korea (Lee and Song, 2013), Morocco (Lau et al., 2016), Pakistan (Naeem and Siddique, 2006), Egypt (Kilany et al., 2016), and Iran (Bahari et al., 2015), but the virus is still present in many locations (Liu et al., 2020). Previous study (Pan et al., 2022) reported that one reason for H9N2 vaccination failure is the high titers of MDAs at the moment of vaccination in one-day-old commercial chickens. Therefore, in this study, we aimed to generate a new vaccine to overcome MDAs interference in chickens.

Turkey herpesvirus (HVT) is considered a potential vector for polyvalent live vaccines in chickens to overcome MDAs interference. Compared to IWV vaccines, some live vector vaccines can stimulate humoral, cellular immunity and even some mucosal immunity against antigens of choice (Swayne et al., 2007; Suarez and Pantin-Jackwood, 2017; Liu et al., 2019). In addition to these advantages of live vector vaccines, a unique characteristic of HVT-based vector vaccines is that they can be minimally or not at all impacted by maternal antibodies. This is probably because the cell-associated nature, the nature of the replication of the HVT vector, and the lack of expression of target antigens on the surface of infected cells or by the recombinant HVT vaccine all contribute to avoiding the MDAs interference against vector and/or target pathogens (Bublot et al., 2007; Faulkner et al., 2013; Bertran et al., 2018). Moreover, HVT-based vaccines have been shown to contain excellent safety characteristics for administration in one-day-old chickens or even for *in ovo* vaccination. In addition, HVT-based vaccines are able to induce lifelong protection against Marek’s disease and the target virus after a single vaccination (Sonoda et al., 2000). However, it is still unclear whether HVT, in the presence of H9N2-specific MDAs, can be used as a vector for H9 HA to induce potent immune responses and prevent transmission of H9N2 AIV in poultry.

When assessing the efficacy of vaccines in the management of infectious diseases, especially zoonotic diseases, the ability to control the transmission of viruses at the population level is important. Transmission of viruses can be assessed by the reproduction ratio (R), the average number of secondary infections caused by a single typical infected animal (Diekmann et al., 1990). In order to stop transmission of a virus, the R value should be smaller than 1. A recombinant virus (rHVT-H9) was generated in our lab and characterized *in vitro* and *in vivo*. We used the SIR transmission model to estimate the R value in chickens vaccinated with the rHVT-H9 in the presence of MDAs. The final MDAs transferred from hens have a high degree of variability in individual broilers (Gharaibeh et al., 2008), which makes the study of MDAs-related vaccination inference more complex. However, a hyperimmune serum that contains mostly IgY has similar isotype proportions to MDAs and therefore can be used to mimic MDAs in specific pathogen-free (SPF) chickens (Hamal et al., 2006; Faulkner et al., 2013). Therefore, in the present study, passively transferred antibodies (PTAs) were used as a model to mimic MDAs in SPF chickens.

## 2. Materials and methods

### 2.1. Ethics statement

All animal studies adhered to the regulation of Care and Use of Laboratory Animals of the Ministry of Science and Technology of the People’s Republic of China, and were approved by the institutional Animal Care and Use Committee of Shanghai Veterinary Research Institute (SHVRI). All experiments involving H9N2 AIV and rHVT-H9 were conducted in the Biological Safety Level 2 (BSL2) facility at the Animal Centre of SHVRI. The permit number was SHVRI-SZ-20200506-01.

### 2.2. Animals and viruses

SPF eggs were purchased from Beijing Merial Vital Laboratory Animal Technology Co., Ltd. and hatched in the laboratory of SHVRI. One-day-old SPF chickens were used in this study. All animals were tagged and housed in high containment isolator (2,200 mm * 860 mm * 1880 mm) and had unlimited access to feed and water.

The low pathogenicity avian influenza virus (LPAIV) H9N2 (A/Chicken/Shanghai/H514/2017) was used in the SHVRI laboratory, abbreviated as H514. The H514 strain is the prevalent strain in China and was isolated and stored by the Research Team of the Etiologic Ecology of Animal Influenza and Avian Emerging Viral Disease, SHVRI. For experimental use, the H514 was propagated in 10-day-old SPF embryonated chicken eggs (ECEs) (Beijing Merial Vital Laboratory Animal Technology Co., Ltd). The virus titers were calculated as median egg infectious doses (EID_50_). The HVT (FC126) parent strain (Okazaki et al., 1970) was stored in liquid nitrogen at the Etiologic Ecology of Animal Influenza and Avian Emerging Viral Disease, SHVRI.

### 2.3. The rHVT-H9 and H9N2 IWV vaccine formation

The H514 HA gene with CMV promoter was cloned into the genome of HVT. The recombinant strain (rHVT-H9) was generated by using an approach as previously described (Kim et al., 1992). In this system, only the target recombinant virus can be generated. The HVT and rHVT-H9 were both propagated separately in CEFs and preserved in liquid nitrogen. Their titers were calculated as PFU in primary CEFs grown and maintained in Dulbecco’s Modified Eagle’s Medium (DMEM) (Biological Industries, BI) supplemented with 1% antibiotics and 10% fetal bovine serum (FBS) (Gibgo, United States), at 37°C with 5% CO_2_ atmosphere.

The H514 (10^9.25^ EID_50_/0.1 ml) was inactivated with 1:2000 β-propiolactone (BPL) by constantly shaking for 16 h at 4°C. The residual β-propiolactone was evaporated at 37°C for 2 h, and then 0.1 ml of the inactivated virus was inoculated in three eggs and incubated for 48 h to confirm the loss of infectivity by a HA assay. The inactivated H514 virus was then mixed with water-in-oil Montanide VG71 (0.85 g/cm^3^) adjuvant (SEPPIC, France) at a volume ratio of 3:7 following manufacturer instructions (Lone et al., 2017). The vaccine was referred to the H9N2 IWV vaccine used in the present study.

### 2.4. Virus growth and plaque assays

The HVT and rHVT-H9 virus growth characteristics were examined by infecting CEFs *in vitro*. CEFs were seeded in 6-well plates (1 × 10^6^/ml/well) and inoculated with 0.01 MOI of the HVT or rHVT-H9 per well. Cells were harvested and titrated at 24, 48, 72, 96, 120 h post-inoculation (p.i). To titrate the replication of those two viruses at different time points, CEFs were seeded in 96-well plates and inoculated with 10-fold dilutions of these viruses in three replications. On 4 days p.i., cytopathic effects were observed and calculated by using PFU.

### 2.5. PCR, western blot and indirect immunofluorescence assay (IFA) analysis

We used PCR, western blot and IFA analysis to identify the expressions of HA proteins in the rHVT-H9. CEFs were seeded in 6-well plates (1 × 10^6^/ml/well) and inoculated with 0.01 MOI of the HVT or rHVT-H9. On 4 days p.i., the cells were harvested for PCR, western blot or IFA.

For PCR, total DNA was extracted from the harvested cells and used for template. Specific primers (Table 1) were used for PCR assay.

**Table 1:** Primes and probes for PCR or real-time PCR.

Gene	Sequence (5′ → 3′)
H9N2-HA-F	CCTTCCTCCAAGACAACGATTAC
H9N2-HA-R	TTGTGGATGTGCAGGAACCAGGC
HVT-F	AGGCCGGGCGAATGGAGATGGTCGACG
HVT-R	GCATGACGGA TCACTAACGA ATTTGCATGTACC
ch-IFN-α-F	CCTTCCTCCAAGACAACGATTAC
ch-IFN-α-probe	TTGTGGATGTGCAGGAACCAGGC
ch-IFN-α-R	AGTGCGAGTGATAAATGTGAGG
ch-IFN-β-F	CCTTGAGCAATGCTTCGTAAAC
ch-IFN-β-probe	CAACGCTCACCTCAGCATCAACAA
ch-IFN-β-R	GGAAGTTGTGGATGGATCTGAA
ch-IFN-γ-F	GTGAAGAAGGTGAAAGATATCATGGA
ch-IFN-γ-probe	TGGCCAAGCTCCCGATGAACGA
ch-IFN-γ-R	GCTTTGCGCTGGATTCTCA
ch-IL-12p40-F	TGGGCAAATGATACGGTCAA
ch-IL-12p40-probe	CTGAAAAGCTATAAAGAGCCAAGCAAGACGTTCT
ch-IL-12p40-R	CAGAGTAGTTCTTTGCCTCACATTTT
ch-β actin-F	TCCCTGGAGAAGAGCTATGAA
ch-β actin-probe	TGGTCAGGTCATCACCATTGGCAA
ch-β actin-R	CAGGACTCCATACCCAAGAAAG

For western blot, the harvested cells were treated with SDS-PAGE loading buffer. The H514 virus collected from embryonated chicken eggs was served as positive control. 10 μl of samples were subjected to SDS-PAGE, and the separated proteins were electroblotted on polyvinylidene fluoride (PVDF) membranes and then blocked with 5% skimmed milk dissolved in 0.5% phosphate-buffered saline with Tween 20 (PBS-T). The membrane was probed with anti-H514 HA monoclonal antibody (2F10) cloned and conserved in the laboratory of Etiologic Ecology of Animal Influenza and Avian Emerging Viral Disease, SHVRI and then anti-mouse IgG-HRP (Sigma, United States). The HA glycoprotein bands were visualized after adding ECL detection reagents.

For IFA, the rHVT-H9 infected CEFs were washed twice with phosphate-buffered saline (PBS) on 4 days p.i. Paraformaldehyde (4%) was added to stabilize cells. The cells were permeabilized using 1% triton and blocked using 1% bovine serum albumin (BSA). The cells were then incubated with 2F10 and then with fluorescence conjugated goat anti-mouse immunoglobulin G (Sigma, United States) at 37°C while protected from light. The results were observed by inverse microscopy (magnifications ×100).

### 2.6. Preliminary animal experiments

Passively transferred antibodies (PTAs) were used as a model to mimic MDAs in SPF chickens as previously described (Pan et al., 2022). Briefly, 0.3 ml of hyperimmune serum with HI titers of 12 log_2_ against H514 were transferred intravenously into a one-day-old SPF chicken. Sera was collected 6 h after PTAs and chickens were vaccinated immediately. Sera was collected weekly after vaccination. One-day-old SPF chickens with PTAs (group 1, *n* = 3) or without PTAs (group 2, *n* = 3) were inoculated with 0.1 ml of the rHVT-H9 (5,000 PFU) subcutaneously in the neck. PBS inoculated chickens with PTAs served as negative control (group 3, *n* = 3). PBS inoculated chickens without PTAs were brought in contact with vaccinated chickens without PTAs (group 4, *n* = 3) to discover whether the vaccine (rHVT-H9) would transmit among chickens. Sera were collected weekly and detected by HI assay.

### 2.7. Transmission animal experiments in chickens with PTAs

Three groups of chickens were used. Each group consisted of 13 one-day-old SPF chickens with PTAs applied in the same way as in the preliminary animal experiments to mimic MDAs. Chickens were subcutaneously inoculated with 0.1 ml of 5,000 PFU rHVT-H9 (group 1), H9N2 IWV vaccine (group 2) or PBS (group 3). Sera were collected weekly and HI titers against H514 were determined by HI assay. Three chickens in each group were sacrificed 28 days after vaccination to identify the replication of the rHVT-H9 in chickens with PTAs and to test the efficacy of cellular and mucosal immunity. Five chickens in each group were challenged with 0.1 ml of 10^6^ EID_50_ of H514 virus intranasally 28 days after vaccination. The other five chickens in each group were added 24 h post-challenge (p.c). Oronasal and cloaca swabs were taken every 2 days until 14 days p.c. At the end of the experiments, all animals were euthanized. The transmission animal experiments design were shown in Table 2.

**Table 2:** Experiment design to quantify the efficacy of the rHVT-H9 in the transmission of H9N2 AIV in chickens with PTAs.

Group	Vaccination	Subgroup	Challenge/dose	Samplings/duration
1	rHVT-H9	Sacrificed (*n* = 3)	None	Feather, spleen and lavage liquids from the nasal cavity and trachea
		Inoculated (*n* = 5)	Intranasal/H514, 10^6^EID_50_	Oronasal and cloaca swabs/ 14 days
		Contact (*n* = 5)	Contact	Oronasal and cloaca swabs/ 14 days
2	H9N2 IWV	Sacrificed (*n* = 3)	None	Feather, spleen and lavage liquids from the nasal cavity and trachea
		Inoculated (*n* = 5)	Intranasal/H514, 10^6^EID_50_	Oronasal and cloaca swabs/ 14 days
		Contact (*n* = 5)	Contact	Oronasal and cloaca swabs/ 14 days
3	PBS	Sacrificed (*n* = 3)	None	Feather, spleen and lavage liquids from the nasal cavity and trachea
		Inoculated (*n* = 5)	Intranasal/H514, 10^6^EID_50_	Oronasal and cloaca swabs/ 14 days
		Contact (*n* = 5)	Contact	Oronasal and cloaca swabs/ 14 days

### 2.8. Hemagglutination inhibition (HI) assay

The antibodies were tested by HI assay as previously described (Suarez et al., 1998). HI titers were determined using the BPL-inactivated H514 virus. Each antigen was diluted to standard 8 HA units in 50 μl. Serum samples were diluted in a series of 2-fold dilutions. 0.5% chicken red blood cells (RBC) in PBS were used in the HI assay.

### 2.9. Cellular and mucosal immunity

An ELISpot assay was performed to measure chicken-interferon-γ (ch-IFN-γ) production by splenocytes of vaccinated chickens with PTAs. Single splenocyte suspension was prepared as previously described and calculated (Ariaans et al., 2008). Briefly, spleen tissue was squeezed through a 70 μm mesh in F12 culture medium (10% FBS) to get a single cell suspension. Ch-IFN-γ ELISpot^PLUS^ kit (HRP) (MABTEGH, Sweden) was used following the manufacturer’s instruction (Ariaans et al., 2009). Briefly, 96-well plates were conditioned by F12 culture medium (Gibco, United States) containing 10% FBS and splenocytes were seeded at 1 × 10^6^ cells/ well in triplicate in F12 medium (10% FBS). The inactivated H514 (50 μl/well) were used to stimulate splenocytes. Con A (10 μg/ml, Solarbio, China) stimulated splenocytes served as positive control. The plates were incubated for 48 h in a 37°C humidified incubator with 5% CO_2_. Ch-IFN-γ was detected by incubation with biotinylated mouse-anti-Ch-IFN-γ (MT&C10-biotin) and Streptavidin-HRP (1:1000). The assay was developed by adding 100 μl of 3-amino-9-ethylcarbazole substrate buffer (Solarbio, China) in the dark until spots appeared. Spots were observed by inverse microscopy (magnifications ×50).

Lavage liquids from the nasal cavity and trachea of the three sacrificed chickens in each group were collected by repeated washing with 0.5 ml of PBS. Indirect ELISA was used to quantify the H514 HA-specific ch-IgA (sIgA) as described (Chen et al., 2017). Briefly, the coating antigen was the purified H514 virus (64 HA/50 μl), and the lavage liquids were used as the test sample. Goat anti-chicken IgA antibody (1:5000 in PBS, Sigma) was used as a second antibody, followed by HRP-linked rabbit anti-goat IgG (1:10,000 in PBS). After three washes with PBS, the plates were overlaid with the *o*-phenylenediamine dihydrochloride (Sigma) substrate. The results were read at 450 nm using a microplate reader.

### 2.10. The rHVT-H9 detection and cytokine mRNA expression in splenocytes

The feathers and spleens of the three sacrificed chickens in the transmission experiments were collected. Splenocytes were prepared as described above. The rHVT-H9 DNA was extracted from chicken feathers and single splenocyte suspension and used for PCR assay to detect the rHVT-H9 strain by using specific primers (Table 1).

Real-time PCR assay was used to detect cytokine mRNA expressions. Total mRNA from splenocytes was extracted using TIANamp Virus RNA kit (TIANGEN, China), and then immediately transcribed to cDNA using primer random 9 and M-MLV reverse transcriptase (Vazyme, China). Real-time RT-PCR assays were performed to quantify the mRNA level of chicken-interferon-α (ch-IFN-α), chicken-interferon-β (ch-IFN-β), chicken-interferon-γ (ch-IFN-γ), and chicken-interleukin-12 protein 40 (ch-IL-12p40) using the resultant cDNA, Universal U Probe Master Mix V2 (Vazyme, China) and specific primers (Table 1). Chicken-β-actin (ch-β-actin) was used as a housekeeping gene. Three independent experiments were conducted at different times. For each gene, the cycle threshold (Ct) values of different treatments at each time point were normalized to the respective endogenous control, ch-β-actin, to get the ∆Ct value. The differences in ∆Ct value between vaccinated and control group (PBS) were calculated (∆∆Ct), Quantification of mRNA levels from each resultant cDNA was expressed as fold changes (2^−∆∆Ct^) (Livak and Schmittgen, 2001).

### 2.11. Detection of virus from oronasal and cloaca swabs

Oronasal and cloaca swabs were collected every 2 days post-challenge and preserved in 0.5 ml of PBS with 10 mg/ml of Penicillin and 10 mg/ml Streptomycin. PBS (0.1 ml) from each sample was inoculated into allantoic cavities of 10-day-old embryonated chicken eggs (ECEs). An HA assay using 0.5% chicken RBC in PBS was done to identify whether these samples contained the virus.

### 2.12. Mathematical models

We used a stochastic SIR model as a model to estimate the transmission of animal experiments described above. As always in the SIR model, all contact animals were defined as “susceptible” (S). All inoculated and any contact animals infected through the course of the experiments were defined as “infectious” (I) from the first day of challenge or when found to be virus positive, until the last day a positive sample was found. After an infected animal stopped shedding the virus, it was defined as “recovered” (R) and thus immune. The total number of animals N = S + I + R. If a contact animal was infected, this was defined as a “case” (C). The reproduction ratio (R) with and without vaccination is the transmission parameter for transmission between individuals (Diekmann et al., 1990). We defined the transmission rate parameter (β) for the transmission rate, βSI/N; the recovery rate parameter (α) for the recovery rate, αI. Transmission-related data (S, I, C) were collected in the transmission experiments and used to estimate the transmission parameters. R was estimated based on the formula: R = β/α. Here, α is the inverse of the average duration of the infectious period T. Hence, R = βT. In this study, chickens in 50/50 ratio (I_0_ = S_0_ = 5) were used in each group to gain the highest power given the experimental size (Velthuis et al., 2007; Sitaras et al., 2016; Tatar-Kis et al., 2020). The design with (S, I) = (5, 5) in two replications for both the vaccinated group and the control group gives sufficient power (60–80% depending on assumptions) to find a significant result when *R* < 1 after vaccination. We used this to quantify the differences between the transmission among rHVT-H9, H9N2 IWV vaccine and PBS vaccinated chickens in the presence of PTAs.

### 2.13. Estimation of transmission parameters and statistical analysis

The probability that a susceptible individual becomes infected during a time interval *Δ*t is given (Velthuis et al., 2007) as:

(1) $$p=1-e^{-\beta \frac{I\Delta t}{N}}$$

The β was estimated using a generalized linear model (GLM) implemented for our analysis in RStudio. In order to get a linear relationship, a complementary loglog link function (ln [−ln (1 − p)]) was used together with the binomial distribution as the error term in the GLM analysis.


(2) $$cloglog\left(p\right)=\ln\left[-\ln\left(1-p\right)\right]=\ln\left(\beta \right)+\ln\left(\frac{I\Delta t}{N}\right)$$

In this relationship, the dependent variable (*p*) is the number of cases (C) divided by the binomial total number of susceptible (S), and the offset equals ln ( $\frac{I\Delta t}{N}$ ). Ln (β), its confidence intervals, and standard error were estimated using the GLM model. Therefore, β was calculated by exponentiation. The other parameter for calculating R is the average infectious period (T) of infected animals, which was directly calculated from the data. The R and its confidence bounds were estimated from the ln (β) and its confidence, calculated by the following equations, assuming independence of ln (β) and ln (T) (Klinkenberg et al., 2002).


(3) Var[ln(R)]=Var[ln(β)]+Var[ln(T)]

The 95% confidence interval will be


(4) $$In\;\left(R\right)\pm 1.96\sqrt{Var\;\left[\;\ln\left(R\right)\right]}$$

The effect of vaccination using the different vaccines was estimated by the same GLM analysis. In this model, we defined β_rHVT-H9_ for chickens immunized with the rHVT-H9, β_IWV_ for chickens immunized with the H9N2 IWV vaccine, and β_PBS_ for chickens inoculated with PBS. The dependent variable was the number of new cases C divided by S (C/S). The dummy variables indicated either the rHVT-H9 group (value 1 or 0) or the H9N2 IWV vaccine group (value 0 or 1). As groups were homogeneous, the regression coefficient c_1_ (see equation below) of the dummy variable for the rHVT-H9 vaccinated chickens gave the extra (or less) transmission in the rHVT-H9 group. This also applied to c_2_, but then for the H9N2 IWV vaccine group. This shows the combined effect of susceptibility and infectivity. Therefore, the equation for the model was:


(5) $$\begin{array}{l} \mathrm{cloglog}\left(p\right)=\mathrm{In}\left(\beta \right)+\mathrm{In}\left(\frac{I\Delta t}{N}\right)=c_{0}+\\ \;\;\;\;\;\;\;\;\;\;\;\;\;\;\;\;c_{1}{\mathrm{Ind}}_{\mathrm{rHVT}-H9}+c_{2}{\mathrm{Ind}}_{\mathrm{IWV}}+\mathrm{In}\left(\frac{I\Delta t}{N}\right) \end{array}$$

(6) $$\mathrm{Herein},\ln\left(\beta \right)=c_{0}+c_{1}{\mathrm{Ind}}_{\mathrm{rHVT}-H9}+c_{2}{\mathrm{Ind}}_{\mathrm{IWV}}$$

Three *β*s were obtained using the estimated regression coefficients from the GLM analysis:


$$\beta_{\mathrm{PBS}}=e^{c0}$$

$$\beta_{\mathrm{rHVT}-H9}=e^{c0+c1}$$

$$\beta_{\mathrm{IWV}}=e^{c0+c2}$$

### 2.14. Statistical analysis

Statistical analyses were performed using GraphPad Prism version 6.0 for Windows (GraphPad Software, San Diego, CA, United States) and SPSS 16 for Windows (SPSS Inc., Chicago, IL, United States). Significant differences were calculated using Student’s *t*-test or Tukey test, posed ANOVA. *p* ≤ 0.05 was considered to be significant.

## 3. Results

### 3.1. Identification and characterization of the recombinant rHVT-H9 *in vitro*

**Figure 1:**
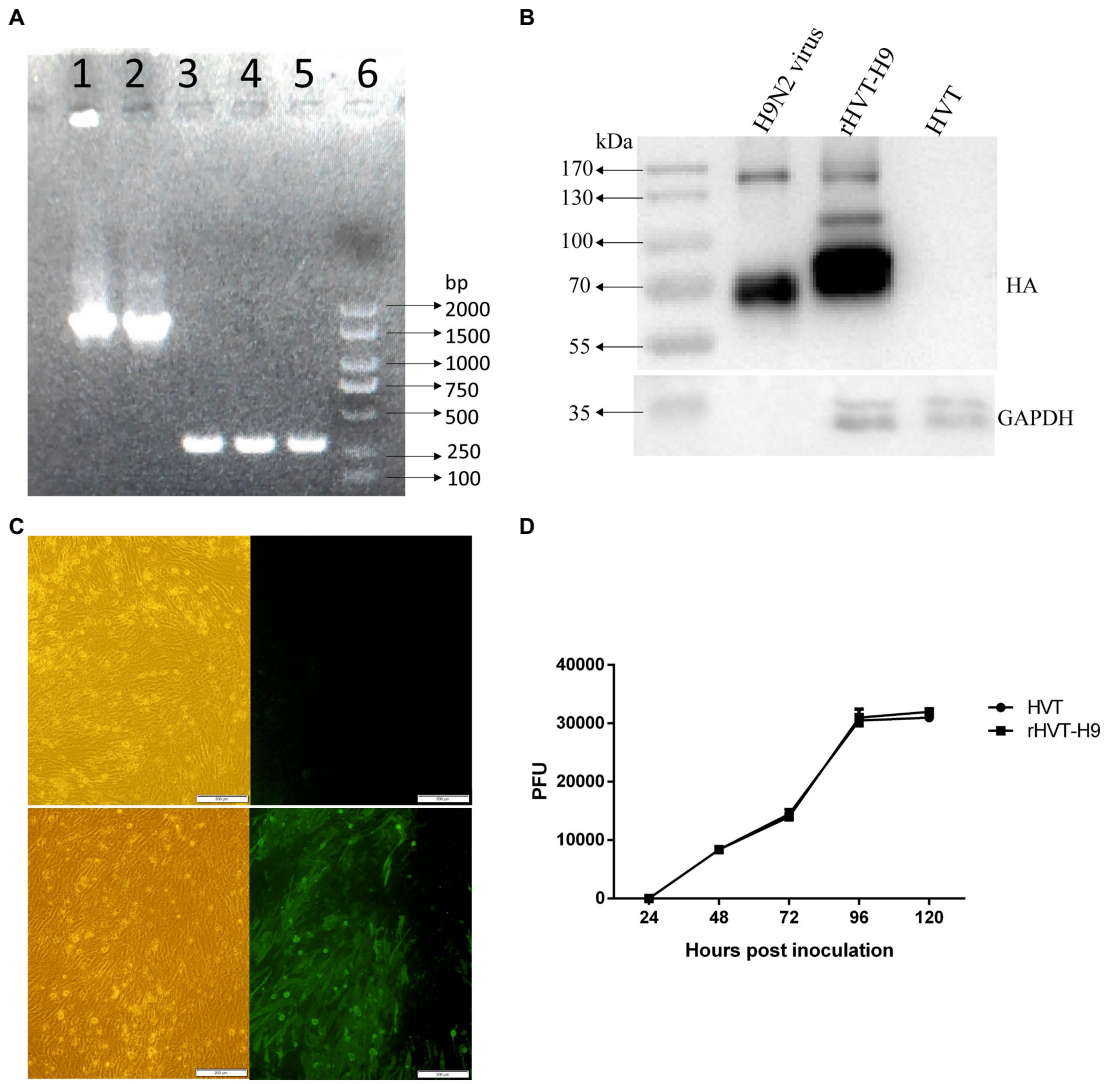
Identification of the rHVT-H9 *in vitro*. **(A)** Gel electrophoresis of the rHVT-H9. Cytopathic CEFs were harvested to examine whether the rHVT-H9 was generated successfully by gel electrophoresis. Line 1 and 2 were amplified by HA-specific primers from the first and second generation of the rHVT-H9 inoculated cells, respectively. Line 3 and 4 were amplified by HVT-specific primers from the first and second generation of the rHVT-H9 inoculated cells, respectively. Line 5 was amplified by HVT-specific primers from the HVT inoculated cells. Line 6 was Marker 2000. **(B)** Western blot analysis of the rHVT-H9. CEFs were inoculated with the rHVT-H9 or HVT virus. Cells were harvested to examine HA proteins expressions by western blot at 4 days p.i. H9N2 (H514) virus collected from embryonated chicken eggs was served as positive control. **(C)** IFA detection of the rHVT-H9. CEFs were inoculated with the rHVT-H9 or HVT as control. Cells were harvested to examine HA proteins expressions by IFA at 4 days p.i. The results were observed by using inverse microscopy (magnifications 100×). The upper was observed on HVT inoculated CEFs; the lower was observed on rHVT-H9 inoculated CEFs. The left was in white light and the right was in blue light **(D)** Growth curve of the rHVT-H9. CEFs were inoculated with the rHVT-H9 or HVT virus with 0.01 MOI. Cells were harvested and titrated at 24, 48, 72, 96 and 120 h p.i. The error bars indicate standard deviations.

To characterize the recombinant rHVT-H9, the rHVT-H9 and parent HVT were inoculated into CEFs. DNA of the rHVT-H9 and HVT was extracted and used for PCR analysis. The agarose and polyacrylamide gels showed that the H9 HA gene was successfully integrated into the HVT vector (Figure 1A). The sequence analysis showed no mutation and the whole recombinant sequences were fully consistent with the expectation.

Western blot and IFA were performed to confirm the expression of HA gene in the HVT vector. CEFs were inoculated with the rHVT-H9 or HVT. Cells were harvested to examine HA expression by western blot or IFA at 4 days p.i. The H514 virus collected from embryonated chicken eggs was used as positive control. The results of western blot showed that the H514 HA proteins, approximately 70 KDa, could be detected in the rHVT-H9 infected CEFs, but not in the HVT infected CEFs. The position of the HA protein was confirmed to be correct by comparison with positive control (Figure 1B). The results of IFA confirmed the H9 HA gene expression in the rHVT-H9 in CEFs (Figure 1C).

Growth kinetics of the rHVT-H9 and parent HVT were analyzed in CEFs. Virus was inoculated into primary CEFs with 0.01 MOI per well in 6 wells-plate, and the inoculated cells were harvested at 24, 48, 72, 96 and 120 h p.i. The harvested cells were titrated into CEFs. The results showed that the recombinant rHVT-H9 had similar growth kinetics to the parent HVT in CEFs (Figure 1D).

### 3.2. Humoral immune response

**Figure 2:**
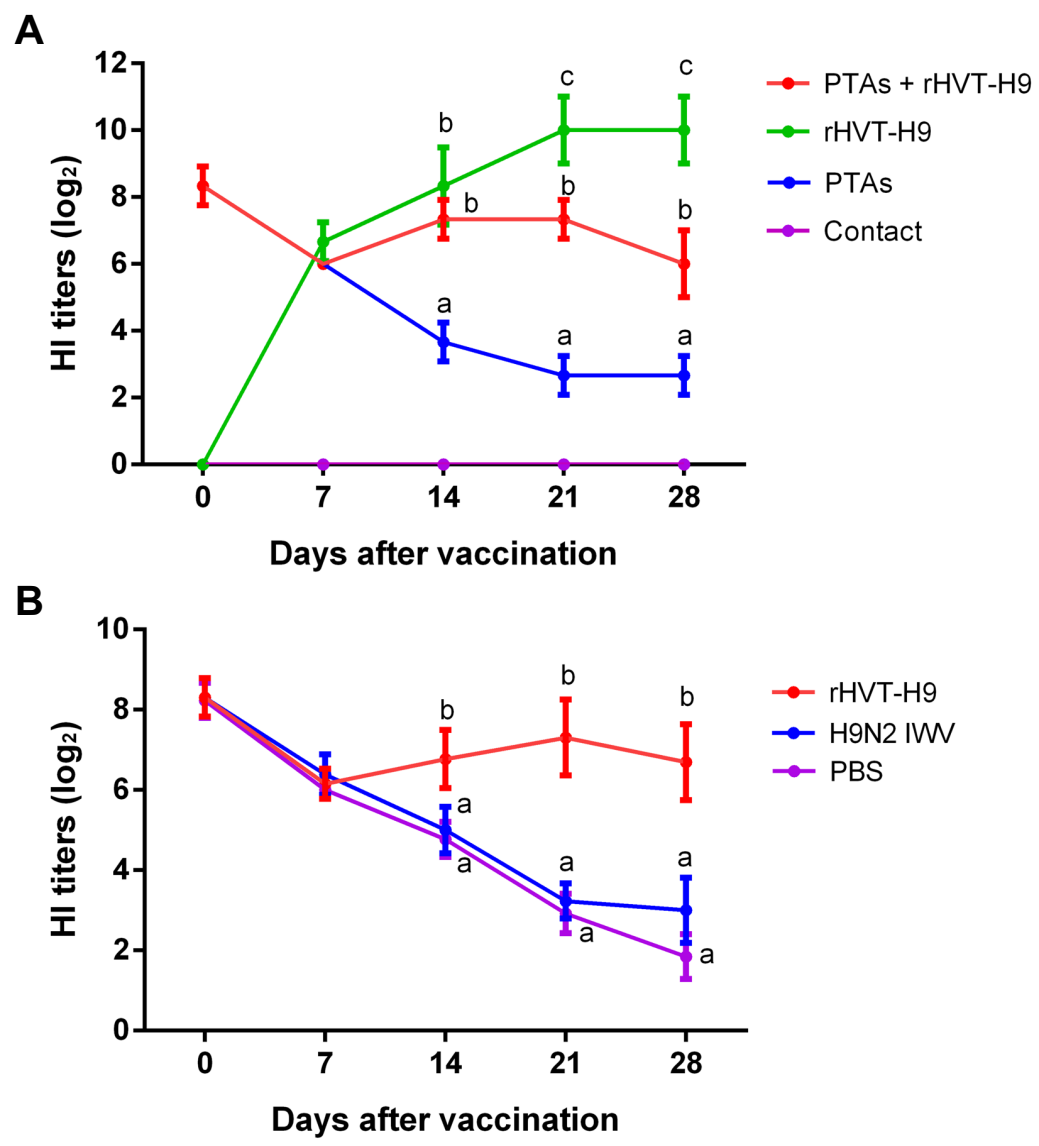
Humoral immune response induced in the preliminary and transmission animal experiment in chickens. **(A)** Sera were collected from the rHVT-H9 immunized chickens with or without PTAs (*n* = 3/group) weekly after vaccination and detected by HI assay. PBS inoculated chickens with PTAs were served as negative control. SPF chickens were contacted with the rHVT-H9 inoculated chickens without PTAs. **(B)** Sera were collected from the rHVT-H9 or H9N2 IWV vaccine inoculated chickens with PTAs (*n* = 13/group) weekly after vaccination and detected by HI assay. PBS inoculated chickens with PTAs were used as negative control. Significant differences were calculated using Tukey test, posed ANOVA. *p* ≤ 0.05 was considered to be significant. The different letters indicate significant differences between the groups at the same time point.

A preliminary animal experiment was performed to assess the safety and immunogenicity of the rHVT-H9 in chickens. The rHVT-H9 was inoculated into chickens with or without PTAs. PBS inoculated chickens without PTA in contact with vaccinated chickens without PTA were used to explore whether the rHVT-H9 could transmit among chickens. Results showed that the vaccinated chickens with PTAs had significantly higher HI titers than PBS inoculated chickens with PTAs, 14 days after vaccination. Vaccinated chickens with PTAs had lower HI titers than vaccinated chickens without PTAs, 21 days after vaccination. Contact SPF chickens without PTAs did not show any HI titers during the experiments (Figure 2A), which indicated that the rHVT-H9 could not transmit among chickens. The results of humoral immune response showed that the HI titers of the rHVT-H9 vaccinated chickens were significantly higher than that of the H9N2 IWV vaccine immunized chickens with PTAs, 14 days after vaccination (Figure 2B).

### 3.3. Cellular and mucosal immune response

**Figure 3:**
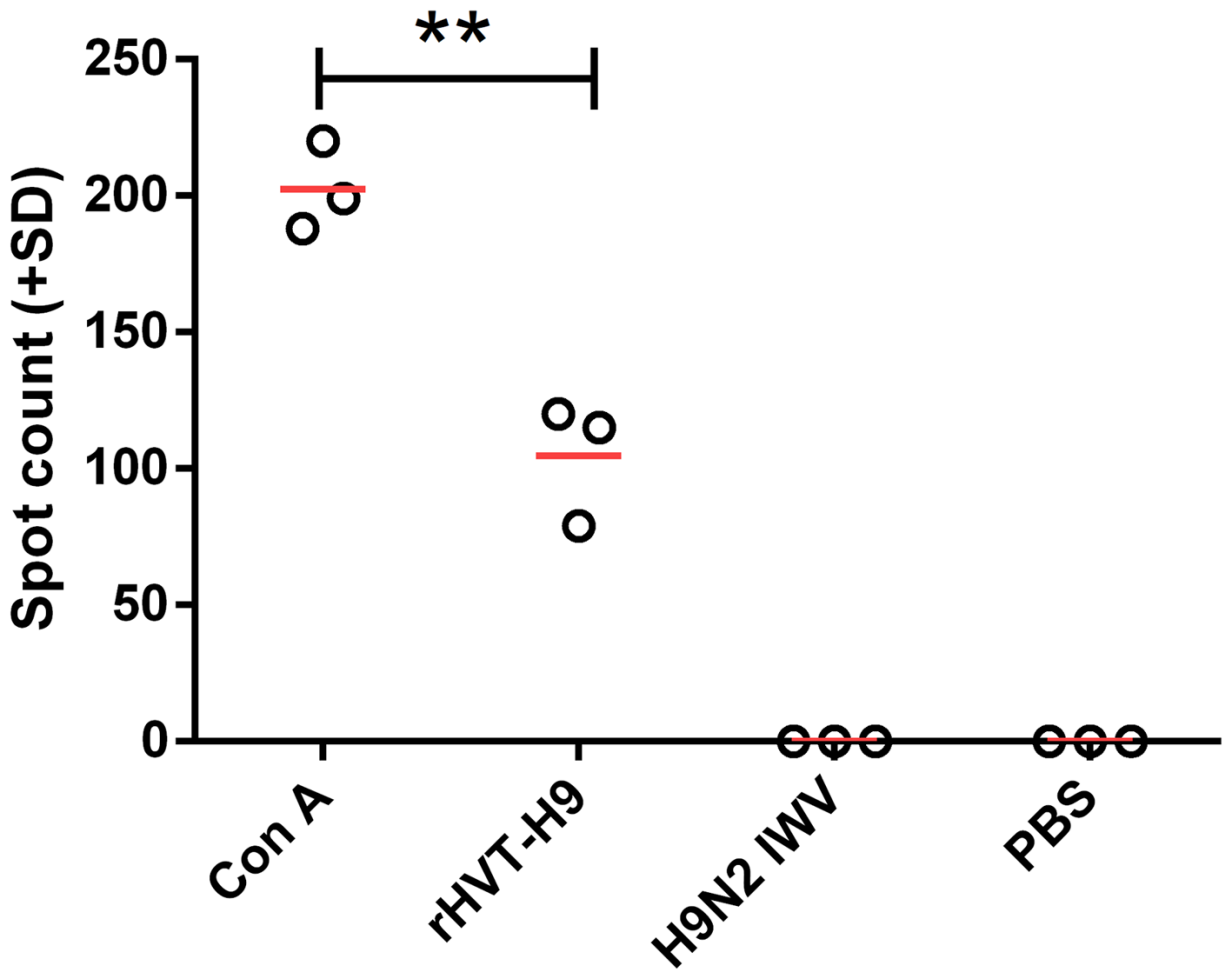
ELISpot assay to measure ch-IFN-γ production by splenocytes of vaccinated chickens with PTAs. Splenocytes of chickens with PTAs (*n* = 3/group) were harvest twenty-eight-days after vaccination. The inactivated H514 (50 μl/well) were used to restimulate splenocytes. Con A (10 μg/ml) stimulated splenocytes served as positive control. Student’s *t*-test was used to compare the differences in means between groups. Symbol (*) denotes differences between two groups (*p < 0.05, **p < 0.01, ***p < 0.001).

To examine the cellular immunity of vaccinated chickens with PTAs, a ch-IFN-γ ELISpot assay was performed following the manufacturer’s instructions (Ariaans et al., 2009). The results showed that the rHVT-H9 induced significantly higher ch-IFN γ secretion than the H9N2 IWV vaccine in chickens with PTAs, which suggested that the rHVT-H9 could induce potential T cell immunity even in the presence of MDAs (Figure 3).

**Figure 4:**
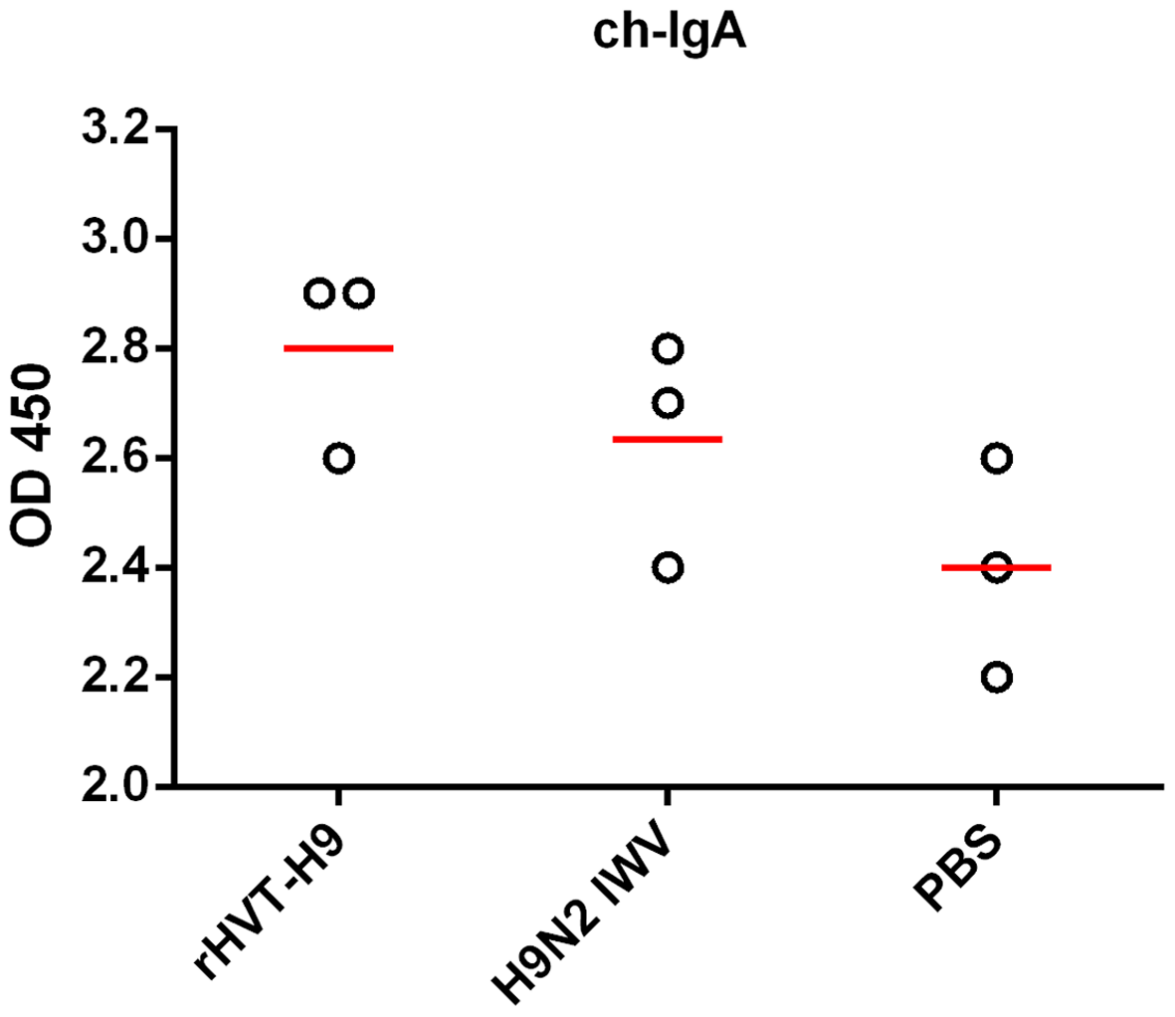
Mucosal immunity after vaccination in chickens with PTAs. Lavage liquids from nasal cavity and trachea of chickens with PTAs (*n* = 3/group) were harvest twenty-eight-day after vaccination and used to quantify H9 HA-specific ch-IgA using indirect ELISA. Significant differences were calculated using Tukey test, posed ANOVA. *p* ≤ 0.05 was considered to be significant.

To assess the efficacy of mucosal immunity, the lavage liquids from the nasal cavity and trachea of the three sacrificed chickens in each group were collected. The indirect ELISA assay showed that. There were no significant differences among all PTAs treatment groups (Figure 4).

### 3.4. The rHVT-H9 detection and cytokines mRNA expression

To identify whether the rHVT-H9 could replicate in chickens with MDAs, three chickens from each group in the transmission experiments were sacrificed 28 days after vaccination, and their feathers and spleens were collected. Total DNA was extracted from feathers and splenocytes for H9 HA-specific PCR assay. The agarose and polyacrylamide gels results showed that all chickens vaccinated with the rHVT-H9 were positive and the positions were as expected in splenocytes (Supplementary Data 1). However, virus was not detected in the feathers of any group.

**Figure 5:**
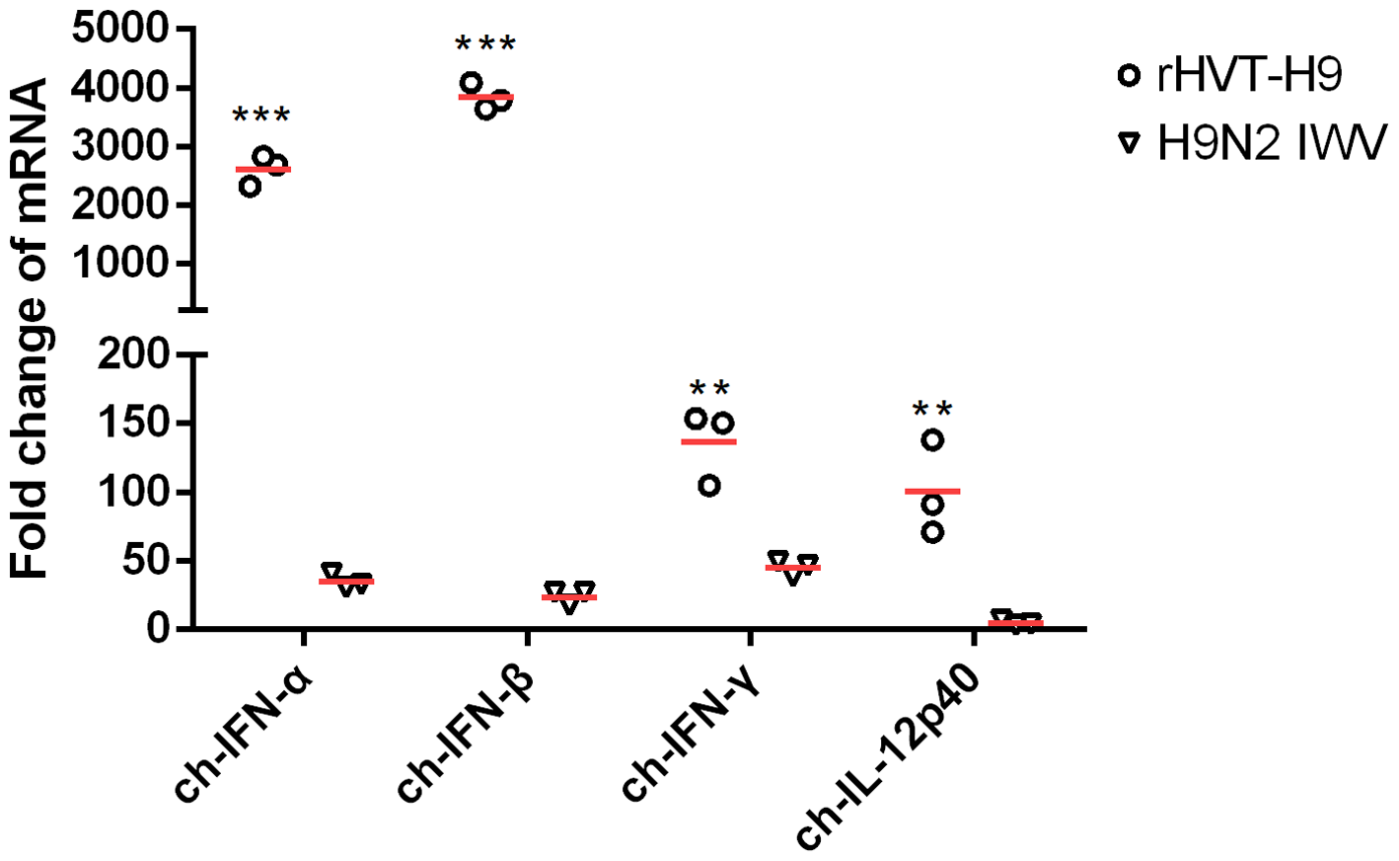
The mRNA expression of cytokines in splenocytes after vaccination in chickens with PTAs. Splenocytes of chickens with PTAs (n = 3/group) were collected twenty-eight-day after vaccination. Real-time PCR assay was used to detect the mRNA expressions of ch-IFN-α, ch-IFN-β, ch-IFN-γ and ch-IL-12p40. Student’s *t*-test was used to compare the differences in means between groups. Symbol (*) denotes differences between two groups (*p < 0.05, **p < 0.01, ***p < 0.001).

Cytokine mRNA expressions in splenocytes were tested by real-time PCR. All cytokines including ch-IFN-α ch-IFN-β ch-IFN-γ and ch-IL-12p40 from the rHVT-H9 vaccinated chickens were significantly higher than those from the H9N2 IWV vaccine immunized chickens with PTAs (Figure 5).

### 3.5. Virus shedding

To identify the virus shedding in the transmission experiments, five chickens from each group (*n* = 10) were challenged, and another five chickens were brought in contact 1 day later. Oronasal and cloaca swabs were taken every 2 days after challenge and were detected in 10-day-old ECEs by HA assay. The results showed that the chickens immunized with the H9N2 IWV vaccine continued shedding virus until 8 days p.c which was similar to PBS inoculated chickens after challenge. However, the rHVT-H9 vaccinated chickens stopped shedding virus earlier, at 4 days p.c. (Table 3).

**Table 3:** Overview of transmission experiment results from Day 1 p.i until Day 10 p.i.

Group	Chicken	Vaccine	Challenge Strain	Day 2	Day 4	Day 6	Day 8	Day 10
1	I	rHVT-H9	H514	+/−	−/−	−/−	−/−	−/−
1	I	rHVT-H9	H514	−/−	−/−	−/−	−/−	−/−
1	I	rHVT-H9	H514	+/+	−/−	−/−	−/−	−/−
1	I	rHVT-H9	H514	+/+	−/−	−/−	−/−	−/−
1	I	rHVT-H9	H514	+/+	−/−	−/−	−/−	−/−
1	S	rHVT-H9	Contact	+/−	−/+	−/−	−/−	−/−
1	S	rHVT-H9	Contact	+/−	−/−	−/−	−/−	−/−
1	S	rHVT-H9	Contact	−/−	−/−	−/−	−/−	−/−
1	S	rHVT-H9	Contact	+/−	−/+	−/−	−/−	−/−
1	S	rHVT-H9	Contact	−/+	−/−	−/−	−/−	−/−
2	I	H9N2 IWV	H514	+/−	+/+	−/−	−/+	−/−
2	I	H9N2 IWV	H514	+/−	+/−	−/−	−/−	−/−
2	I	H9N2 IWV	H514	+/−	+/−	−/−	−/−	−/−
2	I	H9N2 IWV	H514	+/−	+/−	−/−	−/−	−/−
2	I	H9N2 IWV	H514	+/+	−/−	−/+	−/−	−/−
2	S	H9N2 IWV	Contact	−/−	+/−	−/+	−/−	−/−
2	S	H9N2 IWV	Contact	+/−	+/−	−/−	−/+	−/−
2	S	H9N2 IWV	Contact	+/−	−/−	−/−	−/−	−/−
2	S	H9N2 IWV	Contact	+/+	+/+	−/+	−/−	−/−
2	S	H9N2 IWV	Contact	+/−	−/+	−/−	−/−	−/−
3	I	PBS	H514	+/−	+/−	−/+	−/−	−/−
3	I	PBS	H514	+/−	+/−	−/−	+/−	−/−
3	I	PBS	H514	+/+	+/+	−/−	−/−	−/−
3	I	PBS	H514	+/+	+/−	−/−	−/−	−/−
3	I	PBS	H514	+/−	+/−	−/+	−/−	−/−
3	S	PBS	Contact	+/+	+/−	−/+	−/+	−/−
3	S	PBS	Contact	+/−	+/−	+/−	−/−	−/−
3	S	PBS	Contact	+/+	+/+	−/−	−/−	−/−
3	S	PBS	Contact	+/−	+/−	−/+	+/−	−/−
3	S	PBS	Contact	+/−	+/−	+/−	−/+	−/−

Transmission was observed in the rHVT-H9, H9N2 IWV vaccine and PBS inoculated chickens. S, susceptible; I, infectious; +/ or −/ when positive or negative oronasal sample; /+ or /− when positive or negative cloacal samples.

### 3.6. Estimation of transmission rate parameters

The transmission experiment used to quantify the transmission parameters were observed as S, I, C, and N, as described in Table 4.

**Table 4:** Data abstracted from the transmission experiment for parameter estimation for the stochastic transmission model.

Treatment	DS	DE	dt	N	S	I	C
rHVT-H9	1	2	1	10	5	5	4
rHVT-H9	2	4	2	10	1	8	0
rHVT-H9	4	6	2	10	1	2	0
H9N2 IWV	1	2	1	10	5	5	4
H9N2 IWV	2	4	2	10	1	9	1
PBS	1	2	1	10	5	5	5
PBS	2	4	2	10	0	10	0

DS, day of start; DE, day of end; dt, DE-DS; N, total number; S, susceptible; I, infectious; C, new cases.

GLM was used to analyze the data in R studio. The average duration of the observed shedding (T) was directly observed in Table 3. Using Eq. (6), the βs could be calculated. Furthermore, the corresponding R values of the different groups were estimated. The results of all the parameters were shown in Table 5. The average infectious periods were 1.5, 4 and 5.2 in the rHVT-H9, IWV vaccine, and PBS inoculated group, respectively. The *β*s were 1.17 (0.47–3.44), 3.19 (1.11–9.47) and + ∞ (insufficient observations) in the rHVT-H9, IWV vaccine, and PBS groups, respectively. Therefore, R of the rHVT-H9 group was 1.75 (0.71–5.51), smaller than the R 12.76 (4.42–37.88) of the H9N2 IWV vaccine-immunized group and that of the PBS group (0.85- ∞), with estimates based on final size and significance on GLM.

**Table 5:** Transmission rate parameters in different groups.

Group no	Vaccine strain	Challenge strain	β (day-1) (95% CI)	Infectious period (day)	R (95% CI)
1	rHVT-H9	H514	1.17 (0.47–3.44)	1.5	1.75 (0.71–5.15)
2	H9N2 IWV	H514	3.19 (1.11–9.47)	4	12.76 (4.42–37.88)
3	PBS	H514	+ ∞	5.2	+ ∞

## 4. Discussion

MDAs are thought to be one of the reasons for the H9N2 IWV vaccine failure in poultry. To date, few vaccines have been developed to overcome the H9N2-specific MDAs interference in poultry. The rHVT-H9 was characterized in this study. The rHVT-H9 has similar growth kinetics to parent HVT, and the expression of HA proteins were identified by PCR, western blot and IFA *in vitro*. Preliminary animal experiments showed that the rHVT-H9 stimulated excellent humoral immune responses compared to the H9N2 IWV vaccine. The rHVT-H9 did not transmit among chickens, which indicated that it was safe as a vaccine candidate. The transmission animal experiments showed that compared to the conventional H9N2 IWV vaccine, the rHVT-H9 induced high humoral and cellular immunity. Furthermore, the rHVT-H9 inoculated group had a lower reproduction ratio (*R* = 1.75 (0.71, 5.75)) than the H9N2 IWV vaccine and PBS immunized groups in the presence of PTAs (mimicking MDAs) in chickens. The results suggest that the rHVT-H9 can be used as a vaccine candidate to reduce transmission of H9N2 AIV in poultry.

Although MDAs provide early protection for young chickens against various diseases, they also interfere with the efficacy of vaccines. Studies in chickens show that antigen-specific MDAs interfere with the efficacy of the vaccine against H9N2 (Pan et al., 2022), H5N1 (Maas et al., 2011; Abdelwhab et al., 2012), H5N2 (Forrest et al., 2013), infectious bursal disease virus (Naqi et al., 1983) and Newcastle disease virus (NDV; Van Eck et al., 1991). Because MDAs interfere with vaccination efficacy, there will be a period (window) when chickens are susceptible to influenza H9N2 infections, even if a booster vaccination is given at 2–3 weeks. A strength of the study was to design and rescue a new recombinant rHVT-H9 vaccine, which stimulated potential humoral and cellular immunity even in the presence of PTAs (mimicking MDAs) in chickens.

Some live vector vaccines are good options to control pathogens as they not only induce humoral but cellular and sometimes mucosal immunity. On the other hand, some live vector vaccines are sensitive to maternal antibodies against antigens and/or vectors themselves such as Newcastle disease virus (NDV) vector vaccine (Eidson et al., 1976; Lardinois et al., 2016), and fowlpox virus vector vaccine (Swayne et al., 2000; Faulkner et al., 2013). HVT vector vaccines against various pathogens have previously been created to tackle MDAs interference in chickens. Bublot et al. (2007) showed that when using HVT as the vector for infectious bursal disease virus (IBDV), this recombinant virus protects chickens against various IBDV (mild, intermediate and hot) challenge strains despite the high titers of IBD-specific MDAs at the time of vaccination. HVT encoding the HA gene of H5N1 (rHVT-H5) offers 70–90% clinical protection in broilers possessing H5N1 MDAs (Rauw et al., 2012). Bertran et al. (2018) also reported that MDAs to H5N1 have minimal impact on the effectiveness of rHVT-H5. In this study, we found that when compared to the H9N2 IWV vaccine, the rHVT-H9 stimulated significantly higher antibody titers in chickens with PTAs. However, compared to SPF chickens without PTAs, chickens with PTAs had lower antibody titers after inoculation with the rHVT-H9. This indicated that the rHVT-H9 was still slightly hindered by MDAs in chickens. Studies also report that H5N1-specific MDAs interfere with the efficacy of rHVT-H5, compromising and/or delaying the generation of antibodies, although the rHVT-H5 shows protection against clinical signs and reduction of challenge virus shedding with H5N1 highly pathogenic avian influenza (HPAIVs) (Kilany et al., 2015; Suarez and Pantin-Jackwood, 2017).

When assessing the efficacy of a vaccine, the best standard is not only to reduce virus shedding and provide clinical protection, but also to prevent virus transmission. Although many HVT vector vaccines have been reported to overcome MDAs interference in chickens, most only focus on virus shedding and clinical protection rather than virus transmission, possibly leading to continual new infections without these being observed, also known as “silent spread.” This is the first study to quantify the efficacy of the rHVT-H9 on the transmission of H9N2 AIV in chickens with PTAs by using mathematical models. The rHVT-H9 inoculated chickens shed less virus and had a shorter shedding period compared to the H9N2 IWV vaccine and PBS inoculated chickens. Moreover, the R value of the rHVT-H9 inoculated groups was smaller than that of the other two groups but still >1, which indicated that the rHVT-H9 could possibly alleviate but not totally prevent H9N2 AIV transmission in chickens with MDAs. The uncertainty is that MDAs in the field may be more variable and lower than in our experiments. Other researchers showed similar results that MDAs still interfere slightly with the efficacy of HVT-based vaccines (Kilany et al., 2015, Suarez and Pantin-Jackwood, 2017). However, a mathematical model designed by Palya et al. (2018) concluded that the rHVT-H5 vaccination is effective to prevent HPAIV H5N8 transmission in broilers and layers. The different results between their and our study may be because the broilers and layers used in their transmission experiments were selected without influenza-specific MDAs. In our study, chickens contained H9-specific PTAs to mimic MDAs that may interfere with immune responses. The different influenza subtypes, and in addition being highly pathogenic versus (in our case) low pathogenic may also contribute to the difference between the two studies.

T cell-mediated immunity (CMI) and local mucosal immunity are considered to be important against respiratory viruses such as influenza (Kapczynski et al., 2011; Gould et al., 2017). Liu et al. (2019) reported that the recombinant rHVT-H9 stimulated strong CMI and provided full protection against infection of H9N2 AIV in SPF chickens. We found strong CMI as well in the rHVT-H9 inoculated chickens with PTAs by detection of ch-IFN-γ. However, we found that the rHVT-H9 could not provide full protection and prevent transmission of H9N2 AIV in chickens with PTAs, which were not tested in Liu’s study. Insufficient information is available on mucosal immunity in chickens stimulated by HVT-based live vector vaccines. We found that there was no significant difference in mucosal immunity among the rHVT-H9, H9N2 IWV vaccine, and PBS inoculated chickens with PTAs. The weak mucosal immunity may hinder the efficacy of the rHVT-H9 in preventing H9N2 AIV transmission in poultry.

The main strengths of the study are that (1) we are the first to assess the efficacy of the rHVT-H9 in the presence of PTAs; (2) Furthermore, we used SIR model to evaluate the ability of the rHVT-H9 in preventing transmission of H9N2 AIV instead of only infection. The results play a guiding role in clinical application in poultry, suggesting that although the rHVT-H9 is better than the conventional H9N2 IWV vaccine, it still cannot totally prevent transmission of H9N2 AIV in poultry because of the MDAs interference. Therefore, we need to do further studies to improve the rHVT-H9 vaccine.

Two limitations should be noted. First, although we used PTAs to mimic MDAs in SPF chickens, it is still necessary to assess the efficacy of the rHVT-H9 in commercial chickens with real MDAs in poultry since MDAs vary in individual commercial chickens. Second, we collected chickens’ feathers weekly after vaccination, but did not detect any rHVT-H9 in all feather samples. This may be because the parental strain HVT (FC126) is too mild. It is recommended to change to a more virulent strain in future studies.

In summary, we are the first to study the efficacy of the recombinant rHVT-H9 vaccine in reducing the transmission of H9N2 AIV in the presence of PTAs in chickens. The rHVT-H9 stimulated strong humoral and cellular immunity, reducing virus shedding and transmission of H9N2 AIV in the presence of PTAs in chickens but not yet sufficiently since the R value was over 1. Future rHVT-H9 studies should assess the efficacy of rHVT-H9 in commercial chickens in poultry and determine if a booster vaccination with the commercial H9N2 IWV vaccine may totally prevent transmission of H9N2 AIV.

## Data availability statement

The datasets presented in this study can be found in online repositories. The names of the repository/repositories and accession number(s) can be found in the article/Supplementary material.

## Ethics statement

The animal study was reviewed and approved by Animal Care and Use Committee of Shanghai Veterinary Research Institute (SHVRI).

## Author contributions

XP, ZL, and MJ conceived of the study and participated in its design and coordination. QL and SN generated rHVT-H9. XP participated in laboratory work with the help of DH, DY, QT, and XL. XP drafted the manuscript and MJ and QL modified it. ZL, MJ, NB, and MF directed the project. All authors contributed to the article and approved the submitted version.

## Funding

This work was supported by the Shanghai Municipal Science and Technology Major Project (ZD2021CY001), Shanghai Agriculture Applied Technology Development Program, China (2021-02-08-00-12-F00746) and the Agricultural Science and Technology Innovation Project, CAAS, China.

## Conflict of interest

The authors declare that the research was conducted in the absence of any commercial or financial relationships that could be construed as a potential conflict of interest.

## Publisher’s note

All claims expressed in this article are solely those of the authors and do not necessarily represent those of their affiliated organizations, or those of the publisher, the editors and the reviewers. Any product that may be evaluated in this article, or claim that may be made by its manufacturer, is not guaranteed or endorsed by the publisher.
