# Supplementary figures and images for: Efficacy of a recombinant turkey herpesvirus (H9) vaccine against H9N2 avian influenza virus in chickens with maternal-derived antibodies
Source: Front Microbiol. 2023 Jan 26;13:1107975. doi: 10.3389/fmicb.2022.1107975 (PMC9909025; doi:10.3389/fmicb.2022.1107975)

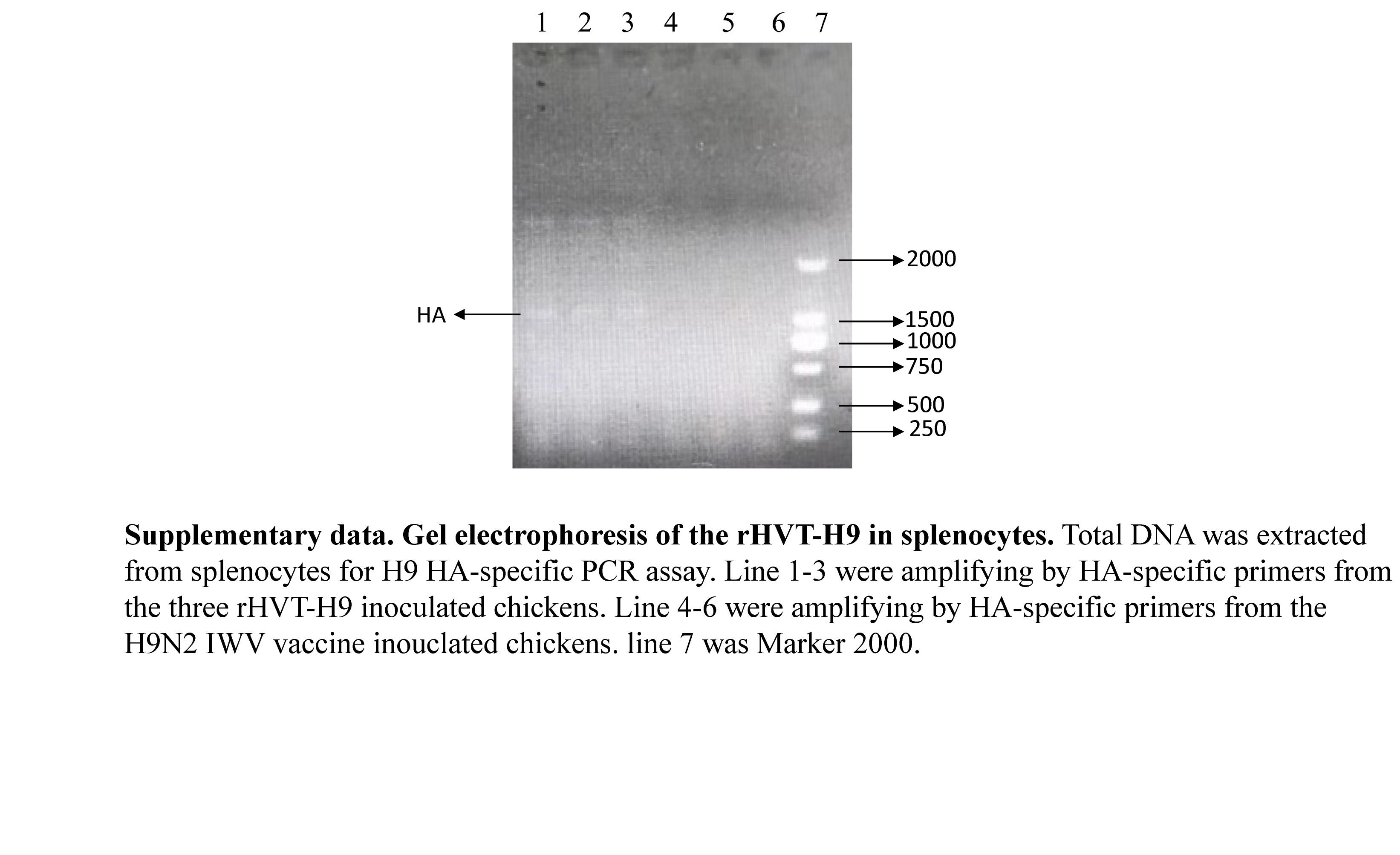

Supplement: Supplementary file 1 [file Image_1.TIF]
